# Deposition of mutant ubiquitin in parkinsonism–dementia complex of Guam

**DOI:** 10.1186/s40478-017-0490-0

**Published:** 2017-11-09

**Authors:** Bert M. Verheijen, Tomoyo Hashimoto, Kiyomitsu Oyanagi, Fred W. van Leeuwen

**Affiliations:** 1 Department of Translational Neuroscience, Brain Center Rudolf Magnus, University Medical Center Utrecht, Utrecht, The Netherlands; 2 Department of Neurology and Neurosurgery, Brain Center Rudolf Magnus, University Medical Center Utrecht, Utrecht, The Netherlands; 3 Department of Neurology, University of Occupational and Environmental Health, Kitakyushu, Fukuoka, Japan; 4 Division of Neuropathology, Department of Brain Disease Research, Shinshu University School of Medicine, Nagano, Japan; 5 Brain Research Laboratory, Hatsuishi Hospital, Chiba, Japan; 6 Department of Neuroscience, Faculty of Health, Medicine and Life Sciences, Maastricht University, Maastricht, The Netherlands

**Keywords:** Parkinsonism-dementia complex, Guam, Mutant ubiquitin, Ubiquitin-proteasome system, TDP-43

Guam parkinsonism–dementia complex (G-PDC) is an enigmatic neurodegenerative disease that affects the Chamorro residents of the Pacific island of Guam. G-PDC is clinically characterized by progressive cognitive impairment with extrapyramidal signs. Pronounced loss of neurons and abundant neurofibrillary tangles (NFTs) are observed throughout the brain of G-PDC patients [6, 7]. Although several hypotheses have been suggested for the cause of G-PDC, notably genetic predisposition and exposure to neurotoxins (e.g., β-*N*-methylamino-L-alanine (BMAA)), the etiology and pathogenesis remain elusive [10].

A frameshift mutant of ubiquitin, known as ubiquitin-B^+1^ (UBB^+1^), was previously found to accumulate in the neuropathological hallmarks of Alzheimer’s disease and several other disorders, including tauopathies and polyglutamine diseases [1, 3, 12] (Fig. 1a-b). UBB^+1^ is a dose-dependent inhibitor of the ubiquitin-proteasome system (UPS) and its accumulation in cells an indicator of protein quality control failure. Impaired protein homeostasis is a frequent feature of neurodegenerative diseases and we hypothesized that accumulation of UBB^+1^ might also be observed in G-PDC. To test whether UBB^+1^ is detectable in G-PDC brains, immunohistochemical analyses were performed on G-PDC post-mortem brain tissue (Table 1). Immunohistochemistry confirmed the presence of numerous NFTs in G-PDC brains [5] (not shown), as well as other pathology that has been described to occur in G-PDC, i.e., TAR DNA-binding protein 43 (TDP-43)-positive inclusions [5] (Fig. 1f-h). Importantly, our results show that UBB^+1^ is present in G-PDC brains. UBB^+1^ deposits were found specifically in cytoplasm of pyramidal neurons and glia (astrocytes in the alveus and stratum oriens) in Ammon’s horn, showing a granular and tangle-like pattern of distribution (Fig. 1c-e). UBB^+1^ was not detected in young control brains (*n* = 2, non-Guamanian cases, ages: 52 and 59 years old) [8]. Aggregate structures containing distinct components of the UPS, i.e., the deubiquitinating enzyme (DUB) ubiquitin C-terminal hydrolase L1 (UCH-L1) [9] (Fig. 1i-k) and the proteasomal ATPase subunit Rpt3/S6b [13] (Fig. 1l-n), were also present in these brains.

**Fig. 1:**
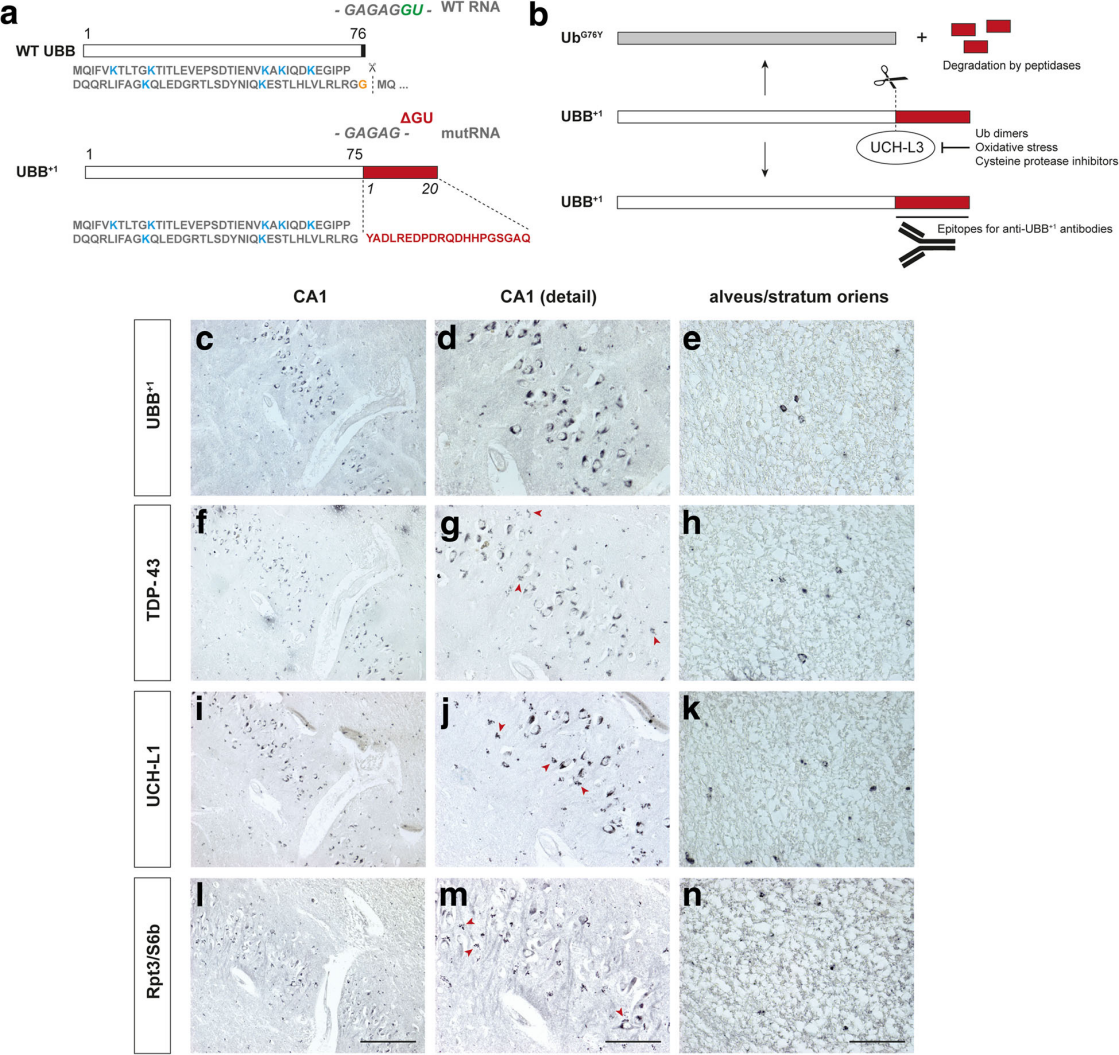
Mutant ubiquitin (UBB^+1^) is deposited in Guam parkinsonism–dementia complex (G-PDC) brains. **a** UBB^+1^ is generated through “molecular misreading”, a type of transcriptional mutagenesis. The resulting unfaithful RNA messengers can generate abnormal proteins with cytotoxic properties. **b** UBB^+1^ contains an extended C-terminal domain, which can be recognized by anti-UBB^+1^ antibodies. Deubiquitating enzymes (DUBs) can hydrolyze this extended C-terminus. However, inhibition of these DUBs, e.g., by oxidative stress conditions, prevents this cleavage, preserving the epitope [2]. **c-e** Immunostaining for UBB^+1^ (Ubi2A, 1:400, Dr. F.W. van Leeuwen [3]) reveals many cytoplasmic structures in neurons and glial cells (i.e., astrocytes in the alveus and stratum oriens) of the hippocampus. **f-h** Additionally, cytoplasmic TAR DNA-binding protein 43 (TDP-43) aggregates can be observed in the same cell types (mouse anti-TDP-43, 1:1000, Abnova). **i-k** Aggregates containing ubiquitin C-terminal hydrolase L1 (UCH-L1) (rabbit anti-UCH-L1, 1:500, Biomol), a DUB, and **l-n** Rpt3/S6b (rabbit anti-Rpt3, 1:400, Biomol), a proteasomal subunit [13], are also found in G-PDC. Several immunoreactive structures show a granular staining pattern (arrowheads). All immunostainings were carried out on 6 μm thick formalin-fixed, paraffin-embedded sections. Panels **c-n** all show representative images of G-PDC hippocampi (adjacent sections from subject #2, Table 1). *Scale bars* 200 μm (**c, f, i, l**), 100 μm (**d, g, j, m**), and 50 μm (**e, h, k, n**)

**Table 1:** Description of the subjects

Subject	Sex	Age of death (years)	Age of onset (years)	Disease duration (months)	Brain weight (g)	Post-mortem delay	Cause of death	UBB^+1^
1	F	51	42	116	850	3 h	perforated gastric ulcer	++
2	M	64	58	72	1275	7 h	pulmonary atelectasis	++++
3	M	52	42	126	1025	4 h	bronchopneumonia	++
4	M	56	46	126	1235	< 10 h	bronchopneumonia	+++
5	F	51	46	59	1135	8 h	bronchopneumonia	++
6	M	84	80	50	1100	14 h	bronchopneumonia	++

This demonstration of UBB^+1^-immunoreactivity and accumulation of particular UPS components in G-PDC brains (*n* = 6) might have important implications for understanding of the pathological mechanisms underlying the disease. UBB^+1^ has previously been shown to induce neuronal defects in in vitro and in vivo experimental models: long-term UPS inhibition due to UBB^+1^ expression causes memory deficits and central breathing dysfunction in mice [4, 8, 11]. In addition, UBB^+1^ might act as a modifier of other pathology in G-PDC. For example, UBB^+1^ may enhance the aggregation and cellular toxicity of the RNA-binding protein TDP-43 through interfering with its degradation. It is striking that UBB^+1^ accumulates in glial cells in G-PDC, because similar glial inclusions have been reported in progressive supranuclear palsy (PSP) [3], a disease that displays some similar topography of neurofibrillary degeneration [10]. Recognition of common mechanistic themes shared by neurodegenerative disorders, such as dysfunctional (ubiquitin-dependent) protein degradation and proteotoxic stress, may help in identifying therapeutic targets that prevent neurodegeneration. It will be interesting to investigate the potential contribution of disrupted proteostasis and UBB^+1^ to G-PDC in more detail in future studies.

## References

[1] de Pril R, Fischer DF, Maat-Schieman MLC, Hobo B, De Vos RAI, Brunt ER, Hol EM, Roos RAC, Van Leeuwen FW (2004). Accumulation of aberrant ubiquitin induces aggregate formation and cell death in polyglutamine diseases. Hum Mol Genet.

[2] Dennissen FJA, Kholod N, Hermes DJHP, Kemmerling N, Steinbusch HWM, Dantuma NP, Van Leeuwen FW (2011). Mutant ubiquitin (UBB^+1^) associated with neurodegenerative disorders is hydrolyzed by ubiquitin C-terminal hydrolase L3 (UCH-L3). FEBS Lett.

[3] Fischer DF, De Vos RAI, Van Dijk R, De Vrij FMS, Proper EA, Sonnemans MAF, Verhage MC, Sluijs JA, Hobo B, Zouambia M, Steur ENHJ, Kamphorst W, Hol EM, Van Leeuwen FW (2003). Disease-specific accumulation of mutant ubiquitin as a marker for proteasomal dysfunction in the brain. FASEB J.

[4] Fischer DF, Van Dijk R, van Tijn P, Hobo B, Verhage MC, van der Schors RC, Li KW, van Minnen J, Hol EM, Van Leeuwen FW (2009). Long-term proteasome dysfunction in the mouse brain by expression of aberrant ubiquitin. Neurobiol Aging.

[5] Hasegawa M, Arai T, Akiyama H, Nonaka T, Mori H, Hashimoto T, Yamazaki M, Oyanagi K (2007). TDP-43 is deposited in the Guam parkinsonism-dementia complex brains. Brain.

[6] Hirano A, Kurland LT, Krooth RS, Lessell S (1961). Parkinsonism-dementia complex, an endemic disease on the island of Guam. I Clinical features Brain.

[7] Hirano A, Malamud N, Kurland LT (1961). Parkinsonism-dementia complex, an endemic disease on the island of Guam. II Pathological features Brain.

[8] Irmler M, Gentier RJG, Dennissen FJA, Schulz H, Bolle I, Hölter SM, Kallnik M, Cheng JJ, Klingenspor M, Rozman J, Ehrhardt N, Hermes DJHP, Gailus-Durner V, Fuchs H, Hrabě de Angelis M, Meyer HE, Hopkins DA, Van Leeuwen FW, Beckers J (2012). Long-term proteasomal inhibition in transgenic mice by UBB^+1^ expression results in dysfunction of central respiration control reminiscent of brainstem neuropathology in Alzheimer patients. Acta Neuropathol.

[9] Lowe J, McDermott H, Landon M, Mayer RJ, Wilkinson KD (1990). Ubiquitin carboxyl-terminal hydrolase (PGP 9.5) is selectively present in ubiquitinated inclusion bodies characteristic of human neurodegenerative diseases. J Pathol.

[10] Steele JC (2005). Parkinsonism-dementia complex of Guam. Mov Disord.

[11] Tan Z, Sun X, Hou F-S, Oh H-W, Hilgenberg LGW, Hol EM, van Leeuwen FW, Smith MA, O'Dowd DK, Schreiber SS (2007). Mutant ubiquitin found in Alzheimer's disease causes neuritic beading of mitochondria in association with neuronal degeneration. Cell Death Differ.

[12] van Leeuwen FW, de Kleijn DPV, van den Hurk HH, Neubauer A, Sonnemans MAF, Sluijs JA, Köycü S, Ramdjielal RDJ, Salehi A, Martens GJM, Grosveld FG, Burbach JPH, Hol EM (1998) Frameshift mutants of beta amyloid precursor protein and ubiquitin-B in Alzheimer's and Down patients. Science. 279;242–247. doi:10.1126/science.279.5348.242 10.1126/science.279.5348.242 9422699

[13] Zouambia M, Fischer DF, Hobo B, De Vos RAI, Hol EM, Varndell IM, Sheppard PW, Van Leeuwen FW (2008). Proteasome subunit proteins and neuropathology in tauopathies and synucleinopathies: consequences for proteomic analyses. Proteomics.

